# Digital self-management application for patients undergoing hemodialysis: influences on health awareness and frailty

**DOI:** 10.1186/s12882-026-05174-8

**Published:** 2026-07-22

**Authors:** Chieko Hamada, Mitsumine Fukui, Yasuhiko Tomino, Reo Kanda, Atsuko Urita

**Affiliations:** 1 Department of Basic Education, Faculty of Health Science and Nursing, Juntendo University, 3-7-33 Omiya, Mishima, Shizuoka, 411-8787 Japan; 2 Tajimabashi Clinic, Medical Corporation SHOWAKAI, Hachiouji, Tokyo, Japan; 3 Medical Corporation SHOWAKAI, Shinjuku-ku, Tokyo, Japan; 4 Division of Nephrology, Ikegami General Hospital, Medical Corporation SHOWAKAI, Ohta-ku, Tokyo, Japan

**Keywords:** Dialysis patients, Self-management, Digital health intervention, eHealth literacy, Patient engagement

## Abstract

**Background:**

Digital health interventions are increasingly used to support self-management in chronic kidney disease; however, their effects on patient-centered outcomes in maintenance hemodialysis (HD) patients remain unclear. This pilot feasibility study investigated the potential impact of a digital self-management application on health awareness, frailty, and eHealth literacy.

**Methods:**

This single-group before-and-after observational study included 12 patients undergoing HD. Participants used a researcher-developed digital self-management application (“eCare”) for 3 months. Clinical parameters, Health Consciousness Scale (HCS) scores, eHealth literacy, and frailty assessed using the Kihon Checklist were evaluated before and after the intervention. Participants were also categorized as active or passive users according to application engagement.

**Results:**

Hemoglobin levels significantly increased after the intervention, whereas other biochemical and dialysis-related parameters remained stable. Total HCS scores significantly changed, with notable improvements in prevention-related awareness and dependence on treatment. Frailty scores showed a trend toward improvement, particularly depressive symptoms. eHealth literacy scores remained stable throughout the study period. Active users demonstrated greater improvements in prevention-related awareness and cognitive function than passive users.

**Conclusions:**

This pilot feasibility study suggests that digital self-management applications may influence health awareness and selected patient-centered outcomes in HD patients, particularly psychological aspects of frailty. Larger controlled multicenter studies are needed to further evaluate the long-term clinical and psychosocial effects of digital health interventions in dialysis care.

**Supplementary Information:**

The online version contains supplementary material available at https://doi.org/10.1186/s12882-026-05174-8.

## Introduction

Patients undergoing maintenance hemodialysis are required to adhere to complex treatment regimens, including strict control of fluid intake, dietary potassium and phosphorus restriction, medication adherence, and management of multiple comorbidities such as cardiovascular disease and diabetes. These demands place a substantial burden on patients and make effective self-management a central component of dialysis care. Poor self-management has been associated with increased hospitalization, complications, and reduced quality of life among dialysis patients [1, 2].

Traditionally, dialysis care has focused primarily on clinical outcomes such as survival and biochemical control. However, recent shifts toward patient-centered care emphasize broader outcomes including well-being, quality of life, and patient engagement in health management. In this context, patients are increasingly expected to actively participate in managing their health and daily life rather than passively following medical instructions [3].

Health literacy plays a critical role in enabling patients with chronic diseases to engage in effective self-management. Health literacy encompasses the ability to obtain, understand, evaluate, and apply health information to make appropriate health decisions. Previous studies have shown that inadequate health literacy is common among patients with chronic kidney disease and dialysis and is associated with poorer health outcomes, reduced treatment adherence, and increased mortality [4, 5].

With the rapid expansion of digital health technologies, electronic health literacy (eHealth literacy) has emerged as an important extension of health literacy. eHealth literacy refers to the ability to seek, evaluate, and apply health information obtained through digital platforms such as the internet, mobile applications, and online health services. Digital health interventions—including mobile health applications, remote monitoring systems, and telehealth programs—have increasingly been introduced to support self-management in chronic diseases [4, 5].

In nephrology, digital health interventions have shown promising potential for improving patient engagement, treatment adherence, and quality of life in individuals with chronic kidney disease and dialysis therapy. Several studies have reported that mobile health applications and digital monitoring systems can enhance patient participation in health management and support behavioral changes necessary for effective self-management [6, 7].

However, engagement with digital health technologies varies widely among patients. Emerging evidence suggests that proactive health awareness, defined as an individual’s active interest in maintaining and improving their health, may be a key determinant of digital health utilization and eHealth literacy. Individuals with higher health awareness are more likely to actively seek health information, critically evaluate digital resources, and apply acquired knowledge to their daily health behaviors [8, 9].

Despite increasing attention to digital health interventions in nephrology, the relationships between health awareness, eHealth literacy, and health-related outcomes in maintenance dialysis patients remain insufficiently understood [9]. In particular, little is known about how these factors relate to frailty status and the acceptability of digital self-management tools among dialysis populations, who are often older and medically complex [10, 11].

Therefore, the present pilot feasibility study aimed to preliminarily investigate the relationships among health awareness, eHealth literacy, and frailty status in patients undergoing maintenance hemodialysis. In addition, this study explored the feasibility and acceptability of a digital self-management application and examined whether digital health interventions may support behavioral changes in health management and patient engagement. By providing preliminary data in a real-world clinical setting, this study may contribute to the future development of larger-scale digital health strategies that support patient-centered self-management in dialysis care.

## Methods

### Study design and participants

**Fig. 1:**
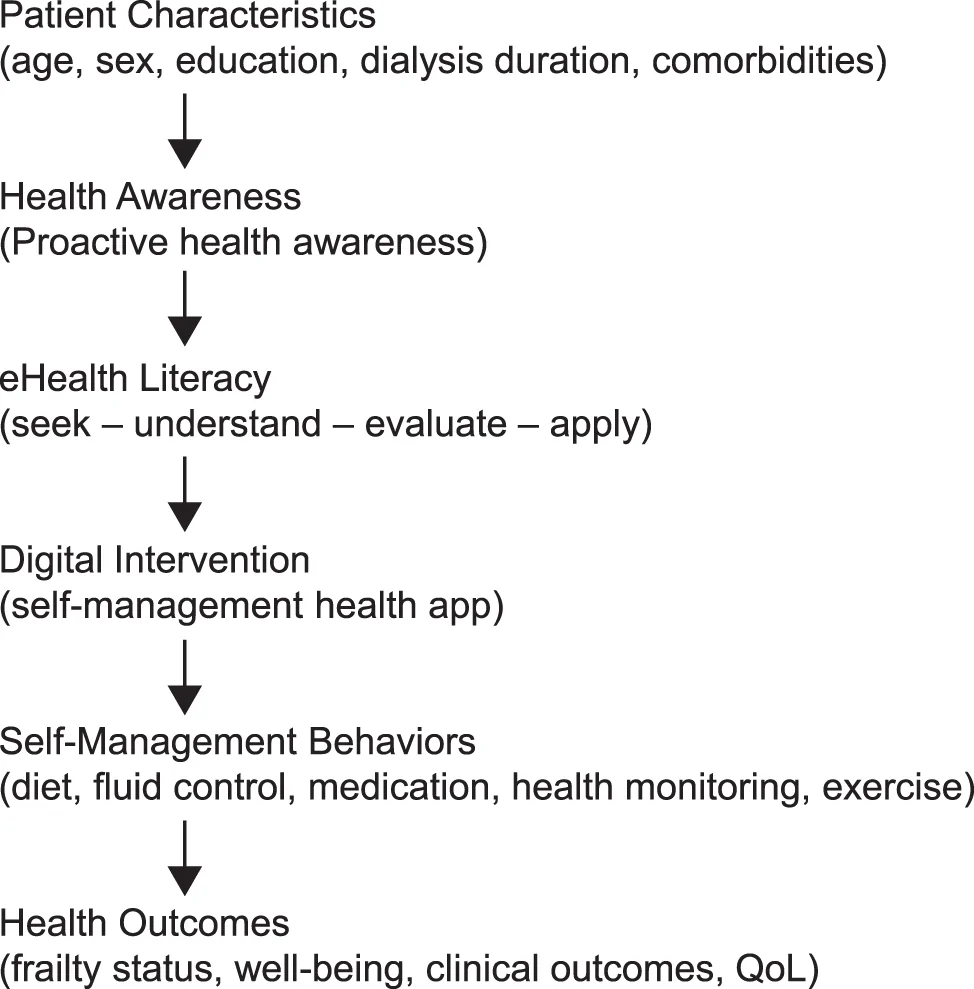
Study design of the present study. Patients undergoing maintenance hemodialysis were assessed at baseline, followed by a 3-month digital self-management intervention using a mobile application. Clinical parameters, health consciousness, eHealth literacy, and frailty status were evaluated before and after the intervention. Subgroup analyses were conducted based on application usage levels

This study was designed as a single-group before-and-after comparative pilot observational study conducted at an outpatient dialysis center. Patients aged ≥ 20 years who had been receiving maintenance hemodialysis three times per week for at least 3 months were eligible for participation. Patients were recruited from outpatient dialysis units during the study period. Individuals who were unable to provide informed consent, had severe cognitive impairment, or had acute medical conditions that could affect participation were excluded. All participants provided written informed consent prior to enrollment (see in Fig. 1).

A health self-management application was used to support participants’ daily health monitoring. The application automatically collected physical activity data, including step counts and the number of flights of stairs climbed, using smartphone sensors. Participants also entered health parameters such as blood pressure and body weight. In addition, weekly health status-including physical condition, sleep quality, dietary intake, and mental well-being-was recorded using a standardized scale. Optional behavioral stamps allowed users to record health-related activities. All collected data were visualized within the application interface on a daily or weekly basis, enabling users to monitor their health status and observe trends over time, thereby supporting self-management behaviors.

### Data collection

Data were collected from medical records, physical assessments, and self-administered questionnaires. Clinical information was obtained from patients’ medical records and included blood biochemical parameters, interdialytic weight gain (IDWG) at the beginning of the week, blood pressure fluctuations during dialysis at the beginning of the week, and the number and types of prescribed medications. These indicators were used to evaluate clinical status and dialysis-related management.

### Physical assessment

Physical function was assessed using grip strength, which is widely used as an indicator of muscle strength and frailty in patients with chronic diseases. Grip strength was measured using a hand dynamometer according to standardized measurement procedures.

### Questionnaire assessments

Participants completed self-administered questionnaires assessing health awareness, digital health literacy, and frailty. Health awareness was assessed using the Japanese version of the Health Consciousness Scale (supplementary material 1) developed by Ozawa et al., which is based on elements of health awareness scales [12, 13] and health motivation scales [12], which are similar concepts to health interest [14]. Digital health literacy was assessed using eHealth Literacy Scale (eHEALS) (supplementary material 2), which evaluates individuals perceived ability to seek, understand, evaluate, and apply health information obtained from electronic sources [15]. Frailty status was assessed using the Kihon Checklist (supplementary material 3a and 3b), a self-reported screening questionnaire developed by the Japanese Ministry of Health, Labor and Welfare that evaluates multiple domains, including physical function, nutritional status, social activity, and cognitive function [16].

### Application-based monitoring

A researcher-developed health self-management application (“eCare”) was used to support daily monitoring during the 3-month intervention period. Physical activity, including step counts and stair climbing, was automatically recorded using smartphone sensors, while blood pressure and body weight were self-reported. Weekly health status, including physical condition, sleep, dietary intake, and mental well-being, was also recorded and visualized within the application, allowing participants to review their own past records. No automated feedback, reminders, educational content, or healthcare provider monitoring functions were included. Healthcare professionals could not directly access participant data through the application. Application usage data included the frequency of body weight and blood pressure entries, weekly health status recordings, and behavioral stamp use. The application was developed specifically for this study by the principal investigator and was not publicly available. Participants were assigned individual IDs, and password management procedures were implemented to reduce the risk of information leakage and protect participant privacy.

### Definition of user engagement groups

Participants were categorized as active or passive users according to application engagement. Active users recorded subjective symptoms or health-related behaviors, whereas passive users relied solely on automatically collected data.

### Ethical considerations

This study was conducted in accordance with the principles of the Declaration of Helsinki. The study protocol was approved by the Institutional Review Board (IRB) Research Ethics Committee of the Faculty of Health Science and Nursing at Juntendo University (IRB approval number 6–005) and the Toyu Medical Research Ethics Review Committee of the Medical Corporation Showakaii (IRB approval number 2024S-13, 2024S-15). Written informed consent was obtained from all participants prior to participation.

### Statistical analysis

Continuous variables are presented as mean ± standard deviation or median (interquartile range), depending on data distribution. Categorical variables are presented as frequencies and percentages. Group comparisons were performed using the Student’s t-test or Mann–Whitney U test for continuous variables and the chi-square test for categorical variables. All statistical analyses were performed using statistical software, and a two-sided *p* value < 0.05 was considered statistically significant.

This study was reported in accordance with the STROBE guidelines.

## Results

### Participants (Table 1)

**Table 1:** Health consciousness scale

Factor 1: Awareness of Health
Qi. I am highly conscious of my health. (Awareness)
Q2. I am interested in information regarding my own health. (Interest in health)
Q3. I pay attention to changes in my health status. (Noticing)
Q4. I am more conscious of my health than people around me. (Comparison with others)
Factor 2: Motivation for Health
Q5. I should devote a certain amount of time to my health. (Time to spend)
Q6. I am willing to spend a certain amount of money on my health. (Expenditure)
Q7. I want to live my life with health as my top priority. (Health first)
Q8. I would do anything to stay healthy. (Sacrifice for health)
Factor 3: Values Regarding Health
Q9. Leisure and hobbies are more important than health. (Priority of hobbies)
Q10. Work and income are more important than health. (Priority of work)
Q11. It is fine to treat a disease after it occurs rather than preventing it. (Indifference to prevention)
Q12. I only worry about my health when I get sick. (Dependence on treatment)

Responses were rated on a four-point Likert scale ranging from “strongly agree” to “strongly disagree,” and were scored from 4 to 1. However, items 9 to 12 of Factor 3 (value placed on health) were reverse scored

The study included 12 patients undergoing maintenance hemodialysis (9 men and 3 women). The mean age was 65.4 ± 6.4 years, and the mean dialysis duration was 5.9 ± 8.1 years. The primary cause of dialysis initiation was diabetic nephropathy in six patients. Regarding medical history, one patient had a history of stroke, two had ischemic heart disease, two had malignant tumors, and one had a history of fracture. The mean number of prescribed medications at baseline was 9.3 ± 3.7 types. In terms of social background, one patient lived alone, three lived with a spouse only, and eight lived with family members. Regarding educational attainment, three patients had graduated from university and nine had graduated from high school. With respect to employment status, one patient was a full-time employee, three were self-employed, one was a non-regular employee, and seven were pensioners. Annual income was less than 4 million yen in six patients and 4 million yen or more in six patients (see in Table 1).

### Clinical outcomes

Changes in clinical parameters before and after the intervention are summarized in Table 2. The mean number of prescribed medications slightly decreased from 9.3 ± 3.7 at baseline to 8.5 ± 3.4 in 3 months, although the difference was not statistically significant. During the study period, hospitalization occurred in four patients; however, these hospitalizations were related to treatment for previously diagnosed malignancy (*n* = 1) and arteriovenous fistula dysfunction (*n* = 3). Interdialytic weight gain at the beginning of the week showed no significant change between baseline and 3 months. Similarly, blood pressure fluctuations during dialysis at the beginning of the week did not significantly change.

**Table 2:** eHealth literacy

1	I know what health resources are available on the Internet.
2	I know where to find helpful health resources on the Internet.
3	I know how to find helpful health resources on the Internet.
4	I know how to use the Internet to answer my questions about health.
5	I know how to use the health information I found on the Internet to help me.
6	I have the skills I need to evaluate the health resources I find on the Internet.
7	I can tell high-quality health resources from low-quality health resources on the Internet.
8	I feel confident in using information from the Internet to make health decisions.

Responses were collected using a 5-point Likert scale: 1 (Strongly disagree), 2 (Disagree), 3 (Neither agree nor disagree), 4 (Agree), and 5 (Strongly agree)

Among the blood biochemical parameters, hemoglobin levels significantly increased from 10.8 ± 1.0 g/dL at baseline to 11.8 ± 1.0 g/dL at 3 months (*p* < 0.05). In contrast, no significant changes were observed in albumin, LDL cholesterol, or inorganic phosphorus levels.

### Health consciousness

The total score of the Health Consciousness Scale significantly decreased from 36.5 ± 7.7 at baseline to 31.8 ± 7.8 at 3 months (*p* < 0.05). Significant changes were observed in several areas, including interest in health (Q2), awareness of health-related issues (Q3), time spent on health-related activities (Q5), indifference toward prevention (Q11), and dependence on medical treatment (Q12). These findings suggest changes in participants’ perceptions and attitudes toward health during the study period (Table 1).

### Frailty status

Frailty status assessed using the Kihon Checklist showed a decrease in the total score from 6.8 ± 4.2 at baseline to 5.4 ± 1.3 in 3 months, although the difference did not reach statistical significance. Among the subdomains, depressive mood showed the greatest reduction (2.0 ± 1.9 vs. 1.0 ± 1.3, *p* < 0.05), suggesting a possible improvement in psychological well-being (Table 1).

eHealth literacy did not show a significant change before and after the use of the digital self-management application.

### Comparison of participant attributes between active and passive users

Baseline characteristics of the study participants are summarized in Tables 3 and 4. No statistically significant differences were observed between the active-use and passive-use groups in age, sex, duration of hemodialysis, primary disease, medical history, number of medications, living arrangements, employment status, or annual income. In contrast, a significant difference was observed in educational background, with a higher proportion of participants with university or college education in the passive-use group compared with the active-use group (*p* < 0.05).

**Table 3:** Frailty Kihon checklist (lifestyle and health questionnaire)

No	Questionnaire Items
1.	Do you go out alone using a bus or train?
2.	Do you go shopping for daily necessities?
3.	Do you manage your own bank deposits and withdrawals?
4.	Do you visit the homes of your friends?
5.	Do you provide advice or consultation to family members or friends?
6.	Do you climb stairs without holding onto a handrail or a wall for support?
7.	Do you stand up from a chair without holding onto anything for support?
8.	Do you walk continuously for about 15 minutes?
9.	Have you fallen in the past year?
10.	Do you have a strong fear of falling?
11.	Have you lost 2–3 kg or more in the past 6 months?
12.	Height: ___ cm, Weight: ___ kg (BMI = ___)
13.	Compared to six months ago, is it harder for you to eat tough/hard foods?
14.	Do you sometimes choke or cough while drinking tea or soup?
15.	Are you bothered by a dry mouth?
16.	Do you go out at least once a week?
17.	Do you go out less frequently than you did last year?
18.	Do people around you tell you that you are forgetful (e.g., “You always ask the same thing”)?
19.	Do you look up phone numbers and make calls by yourself?
20.	Are there times when you do not know today’s date?
In the past two weeks:	
21.	You feel a lack of fulfillment in your daily life.
22.	You are no longer able to enjoy things you used to enjoy.
23.	Things that used to be easy now feel like a burden.
24.	You do not feel like you are a useful person.
25.	I feel tired for no reason.

**Table 4:** Framework of the Frailty Kihon checklist and frailty screening criteria

Framework of the Frailty Kihon Checklist	
#1-5	Lifestyle-related activities (or IADL)
#6-10	Physical functions
#11-12	Nutritional status
#13-15	Oral functions
#16-17	Social withdrawal
#18-20	Cognitive functions
#21-25	Depressive mood
Frailty Screening Criteria	
**A person is identified as "frail" if they meet any of the following conditions:**	
** a. 10 or more items **out of the 20 items (#1–20), excluding the "Depressive Mood" category (#21–25)	
**b. 3 or more items **out of the "Physical Functions" category (#6–10).	
**c. Both items (2 items)** in the "Nutritional Status" category (#11–12).	
**d . 2 or more items** out of the "Oral Functions" category (#13–15).	

### Changes in clinical parameters and questionnaire assessments between groups

Changes in clinical parameters and questionnaire assessments are summarized in Table 5. No significant between-group differences were observed in clinical parameters, including the number of medications, interdialytic weight gain, intradialytic blood pressure changes, or biochemical parameters. Hemoglobin levels increased in both groups, while albumin, LDL cholesterol, and inorganic phosphorus remained relatively stable.

**Table 5:** Principal findings

Number of cases	（Patients）	12
**Gender**	Male: Female	9: 3
**Age**	（years）	65.4 ± 6.4
**Duration of Hemodialysis**	（years）	5.9 ± 8.1
**Primary Disease**	DM: others	6: 6
**Medical History**		
Stroke	（Patients）	1
Ischemic Heart Disease	（Patients）	2
Malignancy	（Patients）	2
Fracture	（Patients）	1
**Number of medications**	（types）	9.3 ± 3.7
**Living arrangements**		12
Living alone	（Patients）	1
Only with spouse	（Patients）	3
Lived with family members	（Patients）	8
**Educational Background**		
University / College	（Patients）	3
High School	（Patients）	9
**Employment Status**		
Full-time employee	（Patients）	1
Part-time employee	（Patients）	1
Self-employed	（Patients）	3
Unemployed	（Patients）	7
**Annual Income (including pension)**		
Less than 4 million yen	（Patients）	6
4 to 8 million yen	（Patients）	5
8 million yen or more	（Patients）	1

Total health consciousness scores and frailty scores decreased in both groups after the intervention, whereas eHealth literacy scores remained stable. In the active-use group, significant improvements were observed in “Indifference to prevention,” “Dependence on treatment,” and the subtotal score for “Values Regarding Health”. Cognitive function also significantly improved in the active-use group. In the passive-use group, “Time spent on health” significantly decreased and use of handrails significantly improved (see Table 6).

**Table 6:** Changes in survey results before and after app implementation

		Baseline	After 3m	P value
Number of medications	（Types）	9.3±3.7	8.5±3.4	np
Hospitalization during the study period	（Patients）	2	4^$^	
Body weight gain^#1^		1.3±0.5	1.2±0.4	np
BP changes^#2^		1.1±0.7	1.5±0.8	np
Biochemical data				
Hb	（g/dL）	10.8±1.0	**11.8±1.0**	**P<0.02**
Albumin	（g/dL）	3.6±0.2	3.7±0.2	np
LDL-C	（mg/dL）	83.1±29.9	84.2±31.4	np
IP	（mg/dL）	4.8±1.2	4.8±1.0	np
Health Consciousness Scale				
Q1. Awareness	（Points）	2.7±1.2	2.6±1.0	np
** Q2. Interest in health**	（Points）	3.3±0.9	**2.8±1.0**	**P<0.05**
** Q3. Noticing**	（Points）	3.2±1.0	**2.8±1.0**	**P<0.05**
** Q4. Comparison with others**	（Points）	2.5±0.8	2.6±0.9	np
** Q5. Time to spend**	（Points）	3.3±0.8	**2.6±1.0**	**P<0.05**
Q6. Expenditure	（Points）	3.1±0.9	2.8±0.7	np
Q7. Health first	（Points）	3.2±1.0	2.8±1.1	np
Q8. Sacrifice for health	（Points）	2.8±0.9	2.6±0.9	np
Q9. Priority of hobbies ^#3^	（Points）	2.6±1.1	2.7±0.9	np
Q10. Priority of work ^#3^	（Points）	3.2±0.7	2.7±0.9	np
** Q11. Indifference to prevention**^#3^	（Points）	3.3±1.0	**2.4±1.1**	**P<0.05**
** Q12.** Dependence on treatment^#3^	（Points）	3.5±0.9	**2.5±1.0**	**P<0.02**
** Total points (48 points)**	（Points）	36.5±7.7	**31.8±7.8**	**P<0.05**
eHealth literacy				
** Total points**	(Points)	20.4±7.0	20.7±6.9	np
Frailty Kihon Checklist				
Lifestyle-related activities（5 items）	（Points）	1.2±1.3	1.4±1.4	np
Physical functions（5 items）	（Points）	1.9±1.7	1.7±1.7	np
Nutritional status（2 items）	（Points）	0.3±0.5	0.3±0.5	np
Oral functions（3 items）	（Points）	0.7±0.9	0.4±0.7	np
Social withdrawal（2 items）	（Points）	0.3±0.5	0.4±0.7	np
Cognitive functions（3 items）	（Points）	0.5±0.5	0.3±0.6	np
** Depressive mood (5 item)**	（Points）	2.0±1.9	**1.0±1.3**	**P<0.05**
Total points（25 points）	（Points）	6.8±4.2	5.4±1.3	np

1: Interdialytic weight gain at the first hemodialysis session of the week: (0: <1.0 kg, 1: 1–3 kg, 2: 3–5 kg, 3: >5 kg)

2: Blood pressure changes during the first hemodialysis session of the week: (0: blood pressure increase, 1: <10 mmHg, 2: 10–25 mmHg, 3: >25 mmHg)

Hb: hemoglobin, LDL-C: low-density lipoprotein cholesterol, IP: inorganic phosphorus

$: Due to scheduled treatment for a previously diagnosed malignancy (1 case) and treatment for arteriovenous fistula dysfunction (3 cases)

#3: Responses were rated on a four-point Likert scale ranging from “strongly agree” to “strongly disagree,” and were scored from 4 to 1. However, items 9 to 12 of Factor 3 (value placed on health) were reverse scored

## Discussion

### Principal findings

In this pilot study of maintenance hemodialysis patients, use of a digital self-management application for 3 months was associated with significant changes in health consciousness and a trend toward improvement in frailty-related status, particularly depressive symptoms. These findings are clinically relevant because patient-centered kidney care increasingly emphasizes outcomes beyond survival alone, including well-being and functional status [17]. Greater application engagement was also associated with more pronounced changes in prevention-related awareness and cognitive function (see Tables 7 and 8).

**Table 7:** Comparison of principal findings between active and passive users

Variable	Unit	Active users	Passive users	p value
**Number of cases**	Male: Female	5: 0	4: 3	np
**Age**	（years）	66.5 ± 5.7	63.9 ± 7.2	np
**Duration of Hemodialysis**	（years）	7.2 ± 10.6	4.2 ± 2.7	np
**Hospitalization within the last year**	（Patients）	1	1	
**Medical History**				
Stroke	(Patients)	1	0	
Ischemic Heart Disease	(Patients)	1	1	
Malignancy	(Patients)	0	1	
Fracture	(Patients)	0	1	
**Number of medications**	（types）	9.3 ± 4.1	9.3 ± 4.1	np
**Living arrangements**				
Living alone	(Patients)	1	0	
Only with spouse	(Patients)	1	2	
Lived with family members	(Patients)	3	5	
**Educational Background**				
University / College	(Patients)	1	2	
High School	(Patients)	4	5	
**Employment Status**				
Full-time employee	(Patients)	1	0	
Part-time employee	(Patients)	0	1	
Self-employed	(Patients)	3	0	
No employee	(Patients)	1	6	
**Annual Income (including pension)**				
Less than 4 million yen	(Patients)	3	3	
4 to 8 million yen	(Patients)	1	4	
8 million yen or more	(Patients)	1	0

**Table 8:** Comparison of clinical outcomes, patient-reported measures and questionnaire assessments between high- and low-application use groups before and after the intervention

Variable	Unit	Active user		Passive user	
		Baseline	3 months	Baseline	3 months
**Number of medications**	(types)	9.3 ± 4.1	9.0 ± 3.4	9.4 ± 3.5	7.8 ± 3.7
**Hospitalization during study period**	(Patients)	1	2	1	2
**Interdialytic weight gain**^**1**^		1.1 ± 0.4	1.0 ± 0.0	1.6 ± 0.5	1.4 ± 0.5
**Intradialytic blood pressure change**^**2**^		1.1 ± 0.7	1.4 ± 0.8	1.0 ± 0.7	1.6 ± 0.9
**Blood biochemical parameters**					
Hemoglobin (Hb)	(g/dL)	11.1 ± 1.3	11.9 ± 1.0	10.4 ± 0.3	11.7 ± 1.0
Albumin	(g/dL)	3.6 ± 0.2	3.7 ± 0.2	3.6 ± 0.2	3.6 ± 0.2
LDL cholesterol (LDL-C)	(mg/dL)	91.6 ± 28.0	94.9 ± 32.9	71.3 ± 31.4	69.2 ± 24.6
Inorganic phosphorus (IP)	(mg/dL)	4.4 ± 1.0	4.6 ± 1.1	5.4 ± 1.4	5.2 ± 0.6
**Health consciousness scale**					
Q5. Time to spent	(points)	3.3 ± 0.8	2.9 ± 1.1	3.2 ± 0.8	2.2 ± 0.8^a^
Q7. Health first	(points)	3.7 ± 0.8	3.0 ± 0.8	2.4 ± 0.9	2.4 ± 1.1
Q10. Priority of work	(points)	3.6 ± 0.5	2.9 ± 1.1	2.6 ± 0.5	2.4 ± 0.5
Q11. Indifference to prevention	(points)	3.9 ± 0.4	2.6 ± 1.3^a^	2.6 ± 1.1	2.2 ± 0.8
Q12. Dependence on treatment	(points)	3.7 ± 0.8	2.1 ± 0.9^a^	3.2 ± 1.1	3.0 ± 1.0
Values Regarding Health (subtotal)	(points)	14.0 ± 1.6	10.6 ± 3.4^a^	14.0 ± 1.6	9.8 ± 2.0
** Total points**	(points)	39.7 ± 6.3	34.4 ± 7.7	32.0 ± 7.6	28.0 ± 6.9
**eHealth literacy**					
** Total points**	(points)	21.9 ± 7.6	22.1 ± 7.3	18.4 ± 6.1	18.6 ± 6.3
**Frailty checklist**					
6.Use of handrail	(points)	0.6 ± 0.6	0.3 ± 0.5	0.8 ± 0.4	0.2 ± 0.4^a^
Cognitive function (subtotal)	(points)	0.4 ± 0.5	0.0 ± 0.0^a^	0.6 ± 0.5	0.6 ± 0.5
** Total points**	(points)	5.3 ± 4.7	4.6 ± 4.7	8.8 ± 2.3	6.6 ± 1.9

Interdialytic weight gain at the first hemodialysis session of the week

Change in blood pressure during the first hemodialysis session of the week

*p* < 0.05 compared with baseline within the same group

### Interpretation of clinical findings

Hemoglobin levels significantly increased after the intervention; however, other biochemical and dialysis-related parameters remained stable. This pattern is consistent with previous CKD digital health studies showing that digital interventions may improve self-monitoring and patient engagement while effects on short-term clinical outcomes remain limited [18]. In addition, causality cannot be inferred because detailed information regarding erythropoiesis-stimulating agent administration and dialysis prescription conditions was not collected.

### Interpretation of health consciousness findings

Changes in the Health Consciousness Scale suggest that visualization and regular monitoring of personal health data may promote reflection and awareness regarding disease management. Active users demonstrated greater improvements in prevention-related awareness and dependence on treatment, suggesting that repeated interaction with the application may support more active engagement in self-management behaviors. In contrast, eHealth literacy scores remained stable, possibly because the application focused primarily on self-monitoring rather than digital health education.

### Frailty and psychological implications

Frailty scores showed a trend toward improvement, particularly depressive symptoms. Frailty is closely associated with functional decline and poor quality of life in dialysis populations [19, 20]. Previous studies have suggested that interventions promoting active participation in care may improve psychological functioning in hemodialysis patients [21]. The improvement in cognitive function observed in active users further supports the potential psychological benefits of sustained application engagement.

### Clinical implications

These findings suggest that digital self-management applications may function primarily as supportive tools that enhance patient awareness, engagement, and psychological well-being rather than rapidly improving biochemical outcomes. Application engagement itself may be an important factor influencing patient-centered outcomes in dialysis care.

### Study limitations

This study has several limitations. The sample size was small, the study was conducted at a single center, and no control group was included, limiting generalizability and causal interpretation. Healthy user bias may also have been present because participants willing to use the application may have had greater motivation toward health management and digital technology use. In addition, the short observation period and lack of detailed data regarding renal anemia management further limit interpretation of the clinical findings. Nevertheless, this study was intentionally designed as an exploratory pilot investigation to evaluate the feasibility, acceptability, and potential multidimensional impact of a digital self-management intervention in a real-world hemodialysis setting. At the time of study planning, evidence regarding the practical implementation and patient acceptance of digital self-management applications among maintenance hemodialysis patients—particularly older individuals with complex comorbidities and frailty-related vulnerabilities—remained limited. Therefore, prior to conducting a randomized controlled trial (RCT), it was considered necessary to obtain preliminary data regarding patient engagement, adherence to app utilization, variability of outcome measures, and the feasibility of longitudinal monitoring in routine dialysis care.

## Conclusions

In conclusion, this pilot feasibility study suggests that a digital self-management application may influence health awareness and selected patient-centered outcomes in maintenance hemodialysis patients, particularly psychological aspects of frailty. Greater application engagement was associated with more pronounced changes in prevention-related awareness and cognitive function. These findings support the potential role of digital self-management tools in patient-centered dialysis care and warrant further evaluation in larger controlled studies.

## Electronic supplementary material

Below is the link to the electronic supplementary material.

Supplementary Material 1

## Data Availability

The datasets generated and/or analyzed during the current study are available from the corresponding author on reasonable request.

## Abbreviations

CKD: Chronic kidney disease
ESKD: End-stage kidney disease
HD: Hemodialysis
HCS: Health Consciousness Scale
eHEALS: eHealth Literacy Scale
KCL: Kihon Checklist
IADL: Instrumental activities of daily living
IRB: Institutional Review Board
RCT: Randomized controlled trial
SD: Standard deviation
