# Supplementary material for: Digital self-management application for patients undergoing hemodialysis: influences on health awareness and frailty
Source: BMC Nephrol. 2026 Jul 22;27:619. doi: 10.1186/s12882-026-05174-8 (PMC13682101; doi:10.1186/s12882-026-05174-8)
Supplement: Supplementary file 1 [file 12882_2026_5174_MOESM1_ESM.docx]

(Supplyment 1)Table 1. Health Consciousness Scale

Factor 1: Awareness of Health

Qi. I am highly conscious of my health. (Awareness)

Q2. I am interested in information regarding my own health. (Interest in health)

Q3. I pay attention to changes in my health status. (Noticing)

Q4. I am more conscious of my health than people around me. (Comparison with others)

Factor 2: Motivation for Health (Table 2)

Q5. I should devote a certain amount of time to my health. (Time to spend)

Q6. I am willing to spend a certain amount of money on my health. (Expenditure)

Q7. I want to live my life with health as my top priority. (Health first)

Q8. I would do anything to stay healthy. (Sacrifice for health)

Factor 3: Values Regarding Health (Table 3a,3b)

Q9. Leisure and hobbies are more important than health. (Priority of hobbies)

Q10. Work and income are more important than health. (Priority of work)

Q11. It is fine to treat a disease after it occurs rather than preventing it. (Indifference to prevention)

Q12. I only worry about my health when I get sick. (Dependence on treatment)

Responses were rated on a four-point Likert scale ranging from “strongly agree” to “strongly disagree,” and were scored from 4 to 1. However, items 9 to 12 of Factor 3 (value placed on health) were reverse scored.

(Supplyment 1)Table 2. eHealth literacy

1. I know what health resources are available on the Internet.
2. I know where to find helpful health resources on the Internet.
3. I know how to find helpful health resources on the Internet.
4. I know how to use the Internet to answer my questions about health.
5. I know how to use the health information I found on the Internet to help me.
6. I have the skills I need to evaluate the health resources I find on the Internet.
7. I can tell high-quality health resources from low-quality health resources on the Internet.
8. I feel confident in using information from the Internet to make health decisions.

Responses were collected using a 5-point Likert scale: 1 (Strongly disagree), 2 (Disagree), 3 (Neither agree nor disagree), 4 (Agree), and 5 (Strongly agree).

(Supplyment 3a)Table 3a. Frailty Kihon Checklist (Lifestyle and Health Questionnaire)

| No | **Questionnaire Items** | |  |
| --- | --- | --- | --- |
| 1. | Do you go out alone using a bus or train? | |  |
| 2. | Do you go shopping for daily necessities? | |  |
| 3. | Do you manage your own bank deposits and withdrawals? | |  |
| 4. | Do you visit the homes of your friends? | |  |
| 5. | Do you provide advice or consultation to family members or friends? | |  |
| 6. | Do you climb stairs without holding onto a handrail or a wall for support? | |  |
| 7. | Do you stand up from a chair without holding onto anything for support? | |  |
| 8. | Do you walk continuously for about 15 minutes? | |  |
| 9. | Have you fallen in the past year? | |  |
| 10. | Do you have a strong fear of falling? | |  |
| 11. | Have you lost 2–3 kg or more in the past 6 months? | |  |
| 12. | Height: ___ cm, Weight: ___ kg (BMI = ___) | |  |
| 13. | Compared to six months ago, is it harder for you to eat tough/hard foods? | |  |
| 14. | Do you sometimes choke or cough while drinking tea or soup? | |  |
| 15. | Are you bothered by a dry mouth? | |  |
| 16. | Do you go out at least once a week? | |  |
| 17. | Do you go out less frequently than you did last year? | |  |
| 18. | Do people around you tell you that you are forgetful (e.g., "You always ask the same thing")? | |  |
| 19. | Do you look up phone numbers and make calls by yourself? | |  |
| 20. | Are there times when you do not know today's date? | |  |
| In the past two weeks: | | | |
| 21. | You feel a lack of fulfillment in your daily life. |  |  |
| 22. | You are no longer able to enjoy things you used to enjoy. |  |  |
| 23. | Things that used to be easy now feel like a burden. |  |  |
| 24. | You do not feel like you are a useful person. |  |  |
| 25. | I feel tired for no reason. |  |  |

(Supplyment 3b)Table 3b. Framework of the Frailty Kihon Checklist and Frailty Screening Criteria

| Framework of the Frailty Kihon Checklist | | |  |
| --- | --- | --- | --- |
| #1-5 | | Lifestyle-related activities (or IADL) |  |
| #6-10 | | Physical functions |  |
| #11-12 | | Nutritional status |  |
| #13-15 | | Oral functions |  |
| #16-17 | | Social withdrawal |  |
| #18-20 | | Cognitive functions |  |
| #21-25 | | Depressive mood |  |
| Frailty Screening Criteria | | | |
| **A person is identified as "frail" if they meet any of the following conditions:** | | | |
|  | **a. 10 or more items** out of the 20 items (#1–20), excluding the "Depressive Mood" category (#21–25) | | |
|  | **b. 3 or more items** out of the "Physical Functions" category (#6–10). | | |
|  | **c.** **Both items (2 items)** in the "Nutritional Status" category (#11–12). | | |
|  | **d.** **2 or more items** out of the "Oral Functions" category (#13–15). | | |
